# Automated Builder and Database of Protein/Membrane Complexes for Molecular Dynamics Simulations

**DOI:** 10.1371/journal.pone.0000880

**Published:** 2007-09-12

**Authors:** Sunhwan Jo, Taehoon Kim, Wonpil Im

**Affiliations:** 1 Department of Chemistry, The University of Kansas, Lawrence, Kansas, United States of America; 2 Department of Molecular Biosciences, Center for Bioinformatics, The University of Kansas, Lawrence, Kansas, United States of America; Temasek Life Sciences Laboratory, Singapore

## Abstract

Molecular dynamics simulations of membrane proteins have provided deeper insights into their functions and interactions with surrounding environments at the atomic level. However, compared to solvation of globular proteins, building a realistic protein/membrane complex is still challenging and requires considerable experience with simulation software. Membrane Builder in the CHARMM-GUI website (http://www.charmm-gui.org) helps users to build such a complex system using a web browser with a graphical user interface. Through a generalized and automated building process including system size determination as well as generation of lipid bilayer, pore water, bulk water, and ions, a realistic membrane system with virtually any kinds and shapes of membrane proteins can be generated in 5 minutes to 2 hours depending on the system size. Default values that were elaborated and tested extensively are given in each step to provide reasonable options and starting points for both non-expert and expert users. The efficacy of Membrane Builder is illustrated by its applications to 12 transmembrane and 3 interfacial membrane proteins, whose fully equilibrated systems with three different types of lipid molecules (DMPC, DPPC, and POPC) and two types of system shapes (rectangular and hexagonal) are freely available on the CHARMM-GUI website. One of the most significant advantages of using the web environment is that, if a problem is found, users can go back and re-generate the whole system again before quitting the browser. Therefore, Membrane Builder provides the intuitive and easy way to build and simulate the biologically important membrane system.

## Introduction

Membrane proteins play important roles in many cellular processes, such as cell signaling [1], [2], translocation of ions and small molecules [3]–[7], energy transduction process [8], [9], and cell-cell recognition [10]. They are quantitatively significant as well. For example, 20–30% of the protein-encoding regions of known genomes encode membrane proteins [11], suggesting that the human genome encodes about 8,000 membrane proteins. Furthermore, about 50% of these membrane proteins are considered putative drug targets [12]. Due to the biological and pharmaceutical importance of membrane proteins, considerable experimental efforts have been made to better understand their structure and function in membrane environments. As a result of technological advances in various fields, more and more membrane protein structures have become available [13], although their numbers are still much smaller than those of globular proteins [14].

Molecular dynamics (MD) simulations of membrane proteins have provided deeper insights into their functions and interactions with surrounding environments at the atomic level [15]–[18]. In particular, when combined with computationally sophisticated and intensive free energy techniques, MD simulations can elucidate the microscopic origins or driving forces of biological functions. Compared to solvation of globular proteins, however, building a realistic protein/membrane complex is still challenging and requires considerable experience with simulation software. The main difficulty in building such a complex system arises from how to insert a protein into a lipid bilayer.

There are two popular methods commonly used to build a realistic protein/membrane complex. In the first method, lipid-like pseudo atoms are first distributed around a protein and then replaced by lipid molecules one at a time [19]–[21]. Individual lipid molecules are randomly selected from a lipid library that contains various conformations of lipid molecules. This method allows one to easily control the system size and the number of lipid molecules while it generates a lipid bilayer nicely packed around the protein. In the second method, a hole is first created in a pre-equilibrated lipid bilayer and then the membrane protein is inserted into the hole [22]–[24]. In general, weak repulsive radial forces perpendicular to the membrane normal are applied to a lipid bilayer until the hole is large enough to accommodate the protein. This method provides a well-equilibrated lipid bilayer, and one might expect less equilibration time than in the first method. For the sake of convenience, the first method is called the “replacement method” and the second method is called the “insertion method” hereinafter. Although both methods are well explained in the literature [19]–[24], considerable efforts and experiences with MD simulation software are required to build a realistic protein/membrane system.

The CHARMM-GUI website (http://www.charmm-gui.org) provides a graphical user interface (GUI) of a collection of modules that helps users to setup their MD simulations in a web browser. Membrane Builder, one of the modules in CHARMM-GUI, aims to help users to build a sophisticated protein/membrane system easily and interactively in a web browser through a generalized and automated building process including system size determination as well as generation of lipid bilayer, pore water, bulk water, and ions. The main idea is to let users define various parameters, such as the system size and shape, ion concentration, lipid type, and bilayer generation method in a full GUI fashion, and to provide the generated complex structure and input files. However, default values that were elaborated and tested extensively are given in each step to provide reasonable options and starting points for both non-expert and expert users. We have further developed and generalized both replacement and insertion methods to adopt them in Membrane Builder to build a membrane bilayer efficiently.

In this paper, we describe and illustrate the generalized protein/membrane complex building process. Its efficiency has been examined by building a total of 90 protein/membrane complexes of 12 transmembrane and 3 interfacial membrane proteins, that have considerably different shapes and sizes, with three types of lipid bilayers (dimyristoylphosphatidylcholine (DMPC), dipalmitoylphosphatidylcholine (DPPC), and palmitoyloleoylphosphatidylcholine (POPC)) and two different system shapes (rectangular and hexagonal). Each equilibrated system is available at the archive on the CHARMM-GUI website.

## Results

### Generalized Protein/membrane Complex Building Process

**Figure 1:**
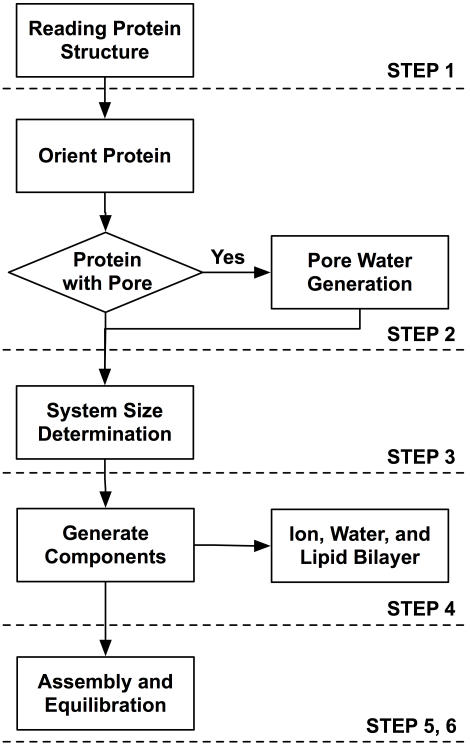
Overview of generalized process of building protein/membrane complex system.

As shown in Fig. 1, the overall process to build a protein/membrane complex has been generalized and automated in six subsequent steps. Each step was designed to incorporate users' specific options through a web browser, and generate and run CHARMM input files. Individual input and output files including generated structures as well as an archive of all the created files are available in each step. One can visualize the generated system in each step so that, if necessary, one can go back to the previous step and modify the options interactively. Video demonstrations on how to use Membrane Builder (“Membrane Builder Lecture” and “Membrane Builder Demo”) are available on the CHARMM-GUI website.

#### Step 1–PDB Reading

The objective of this step is to read a protein structure, manipulate it if necessary, and generate a structure topology file. Users can upload their own pre-oriented protein structure, or specify PDB entry ID and a database, either of PDB database [14] or OPM database [25]. Protein structures from OPM database are pre-oriented with respect to the membrane normal (the *Z*-axis by definition), so that one may use them without any modification of the protein orientation (see Step 2 below). In this step, users can select a model structure (in the case of NMR ensemble structures) or a part of residues in each chain. After the model and chains are selected, users can rename or remove engineered residues, choose terminal groups for each chain, protonate and/or phosphorylate residues, and make disulfide bond between two residues.

#### Step 2–Orient Protein

In general, a membrane protein structure from PDB database [14] does not have proper information on relative disposition of the protein in a membrane bilayer. In Membrane Builder, one can place the protein appropriately in a lipid bilayer by aligning its principal axis or a vector between two specific C_α_ atoms along the *Z*-axis, by translating the protein along the *Z*-axis, and by rotating it around the *X*-axis. It is assumed that the membrane normal is parallel to the *Z*-axis and its center is located at *Z = *0 Å. Alignment with the principal axis is useful for the proteins that have a symmetric shape. The usage of a vector defined by specific two residues is useful for irregular-shaped proteins if one can identify two residues that are parallel to the *Z*-axis. In the case that the protein structure is not easily aligned with the available options, one should align it in a local machine and then upload it. One can skip the alignment step for the protein structures from OPM database [25] or pre-oriented structures.

**Figure 2:**
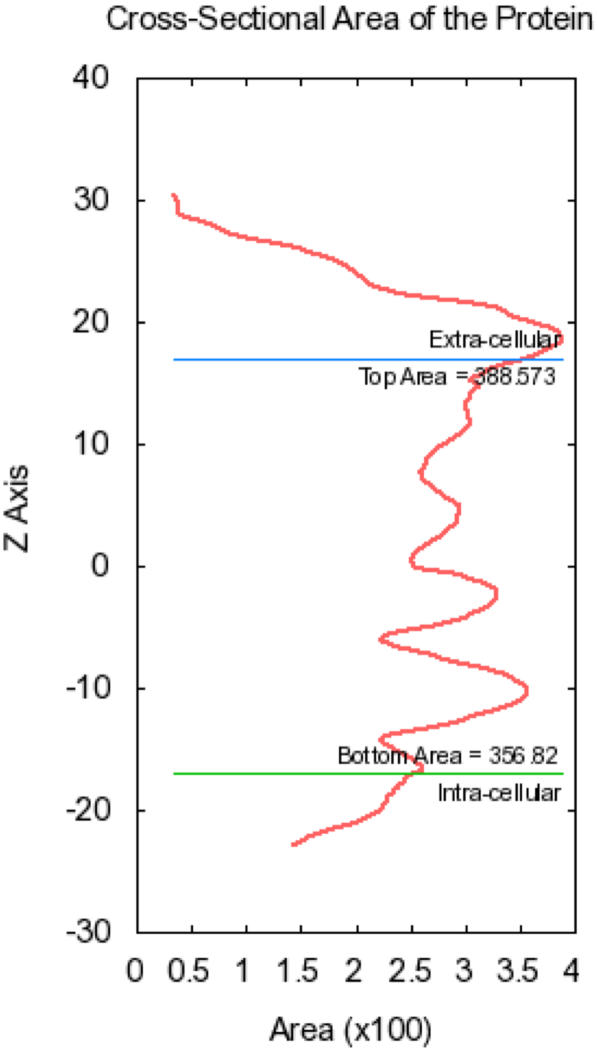
Cross-sectional area profile of PDB:2HAC along the *Z*-axis.

After alignment, Membrane Builder generates pore water, if specified, and calculates the protein cross-sectional area along the *Z*-axis. We have developed a new method to generate water molecules inside pores with any kind of shapes and numbers (see Pore Water Generation for details). The cross-sectional area profile is used to determine protein areas that correspond to the maximum values at 10 Å<*Z*<20 Å and −20 Å<*Z*<−10 Å, respectively. Figure 2 shows the calculated profile for PDB:2HAC (ζ-ζ transmembrane dimer). The protein areas in the top and bottom lipid leaflets are used to determine the system size in the next step. The cross-sectional area of pore water, if generated, is incorporated into the protein area so that the system size in the *XY* plane is determined as the sum of the protein area and the lipid area (see below).

#### Step 3–Determine System Size

**Figure 3:**
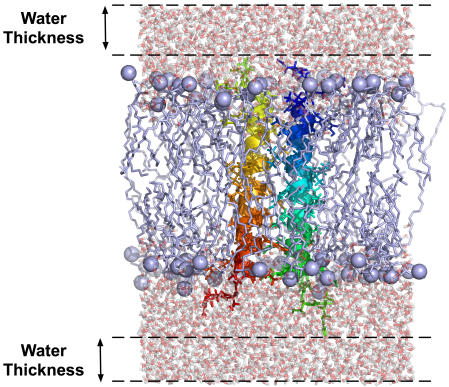
Thickness of bulk water from the protein extent (PDB:2HAC) along the *Z*-axis.

There are currently three types of lipid molecules available to build homogenous lipid bilayers, such as DMPC (experimental cross-sectional area of 60.7 Å^2^ at 303 K), DPPC (64.0 Å^2^ at 323 K), and POPC (68.3 Å^2^ at 303 K) [26]. By using the protein areas determined in the previous step and the experimental cross-sectional areas of lipid molecules, one can estimate the number of lipid molecules to build a system with specific dimension and shape (rectangular or hexagonal) in the *XY* plane, or vice versa. Membrane Builder facilitates this process by determining the system size based on users' inputs for (1) number of lipid layers, (2) number of lipid molecules, or (3) system dimensions. The number of lipid layers corresponds to the number of lipid molecules from a protein to the closest system edge so that this option can be used to quickly estimate the total number of lipid molecules. For example, “1.5 lipid layers” means, at least, three lipid molecules will be placed between two proteins in the primary and image systems. The system size along the *Z*-axis is determined by specifying the thickness of bulk water from the protein extent along the *Z-*axis, as shown in Fig. 3. In the case of membrane interfacial proteins whose extents do not cover the lipid bilayer, a minimum protein extent is set to *Z* = 20 Å and *Z* = −20 Å.

**Figure 4:**
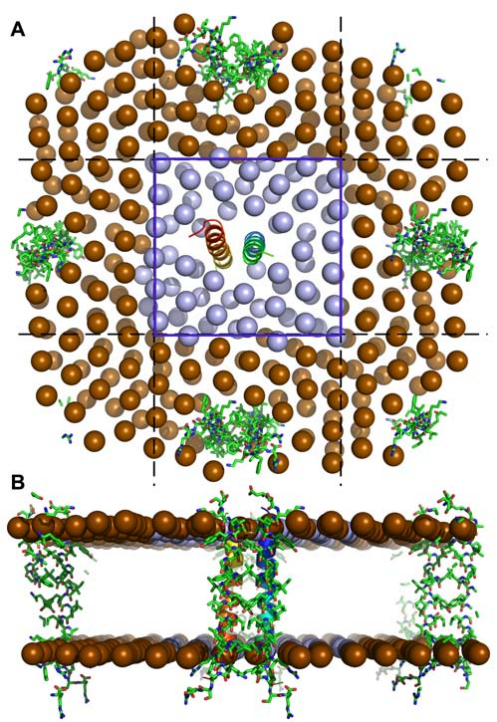
Packing image with lipid-like pseudo atoms around PDB:2HAC. An option of 1.5 lipid layer was used to estimate the number of lipid molecules. Both primary (blue region) and image systems (yellow region) in *XY* plane are visualized to provide a general idea about the lipid packing in the protein/membrane complex.

After the system size determination is completed, Membrane Builder provides a detailed summary of determined system size and a possible lipid-packing image to help users to check if the system size is proper. Figure 4 shows a packing image of PDB:2HAC, which corresponds to a configuration generated by MD simulations of lipid-like pseudo atoms whose cross-sectional area is similar to the lipid molecule. The image systems are also displayed to provide a better idea about the system size. If the system is smaller or larger than expected, users can go back and specify the numbers of lipid molecules (option 2) or system dimensions (option 3) to enlarge or reduce the system size.

#### Step 4–Build Components

Based on the system size information, Membrane Builder generates individual components to fully solvate the protein, such as a lipid bilayer, bulk water, and counter ions. If pore water molecules were generated in Step 2, users can refine them in this step to make sure that there is no water molecule left outside of the protein in the membrane hydrophobic core region (see Pore Water Generation for details). If the ion concentration (*C*) is specified, the numbers of KCl ions (*N*_K_ and *N*_Cl_) are determined by the ion-accessible volume (*V*) and the total charge of the system (*Q*_sys_), i.e., *N*_K_ = *C*·*V*−*Q*_sys_/2 and *N*_Cl_ = *C*·*V*+*Q*_sys_/2, to neutralize the system charge. The initial configuration of ions is then determined through Monte Carlo simulations using a primitive model, i.e., scaled Coulombic and van der Waals interactions. Note that only monovalent K and Cl ions are currently available, but more ion types can be incorporated easily upon users' request. It is this step that users select the lipid bilayer generation method, i.e., the insertion method or the replacement method (see below for details).

#### Step 5, 6–Assemble Components and equilibrate the system

**Figure 5:**
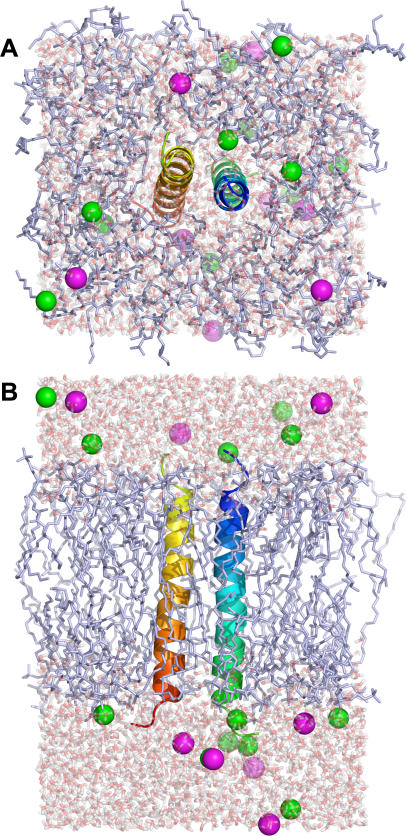
Assembled PDB:2HAC/DMPC structures with water thickness of 10 Å (see Figure 3) and 0.15 M of KCl. (A) top and (B) side views. Green and magenta spheres represent chloride and potassium ions, respectively.

Each component that has been generated in the previous step will be assembled in this step as shown in Fig. 5. One of the most significant advantages of using the web environment is that, if a problem is found, users can go back and re-generate the whole system again before quitting the browser. Therefore, the visualization of the initially assembled structure is important to verify if the system is reasonable.

After the protein/membrane complex is assembled, the equilibration must be performed to relax the uncorrelated initial system before MD production simulations. However, due to the significant amount of computing power that cannot be hosted in our server, Membrane Builder provides six consecutive CHARMM input files for equilibration, which can be modified for continual production simulations. As shown in Table 1, to assure gradual equilibration of the initially assembled system, various restraints are applied to the protein, water, ions, and lipid molecules during the equilibration [27]: (1) harmonic restraints to ions and heavy atoms of the protein, (2) repulsive planar restraints to prevent water from entering into the membrane hydrophobic region, and (3) planar restraints to hold the position of head groups of membranes along the *Z*-axis. These restraint forces are slowly reduced as the equilibration progresses. To warrant the successful equilibration, i.e., to avoid instability of dynamics integrations during equilibration, the NVT dynamics (constant volume and temperature) is used for the first and second steps, and the NPAT (constant pressure, area, and temperature) dynamics for the rest at 303.15 K (DMPC and POPC) and 323.15 K (DPPC).

**Table 1:** Detailed information on each equilibration step

Step	Ensemble^1^	Timesteps	Equilibration Time	Force Constants for Harmonic Restraint^2^				
				Protein Backbone^3^	Protein Sidechain^3^	Water^4^	Lipid^5^	Ion^3^
1	NVT	1 fs	25 ps	10.0	5.0	2.5	2.5	10.0
2	NVT	1 fs	25 ps	5.0	2.5	2.5	2.5	0.0
3	NPAT	1 fs	25 ps	2.5	1.0	1.0	1.0	0.0
4	NPAT	2 fs	100 ps	1.0	0.5	0.5	0.5	0.0
5	NPAT	2 fs	100 ps	0.5	0.1	0.1	0.1	0.0
6	NPAT	2 fs	100 ps	0.1	0.0	0.0	0.0	0.0

1. NVT stands for constant volume and temperature, and NPAT for constant pressure, area, and temperature.

2. Force constants are in kcal/(mol·Å^2^).

3. Positional harmonic restraints.

4. Harmonic restraints to keep water molecules away from the membrane hydrophobic region.

5. Harmonic restraints to keep the lipid tail in –5 Å<*Z*<5 Å, and lipid head groups close to the membrane surface (*Z* = ±17 Å for DMPC and *Z* = ±19 Å for DPPC and POPC).

### Lipid Bilayer Building Methods: Insertion and Replacement

**Figure 6:**
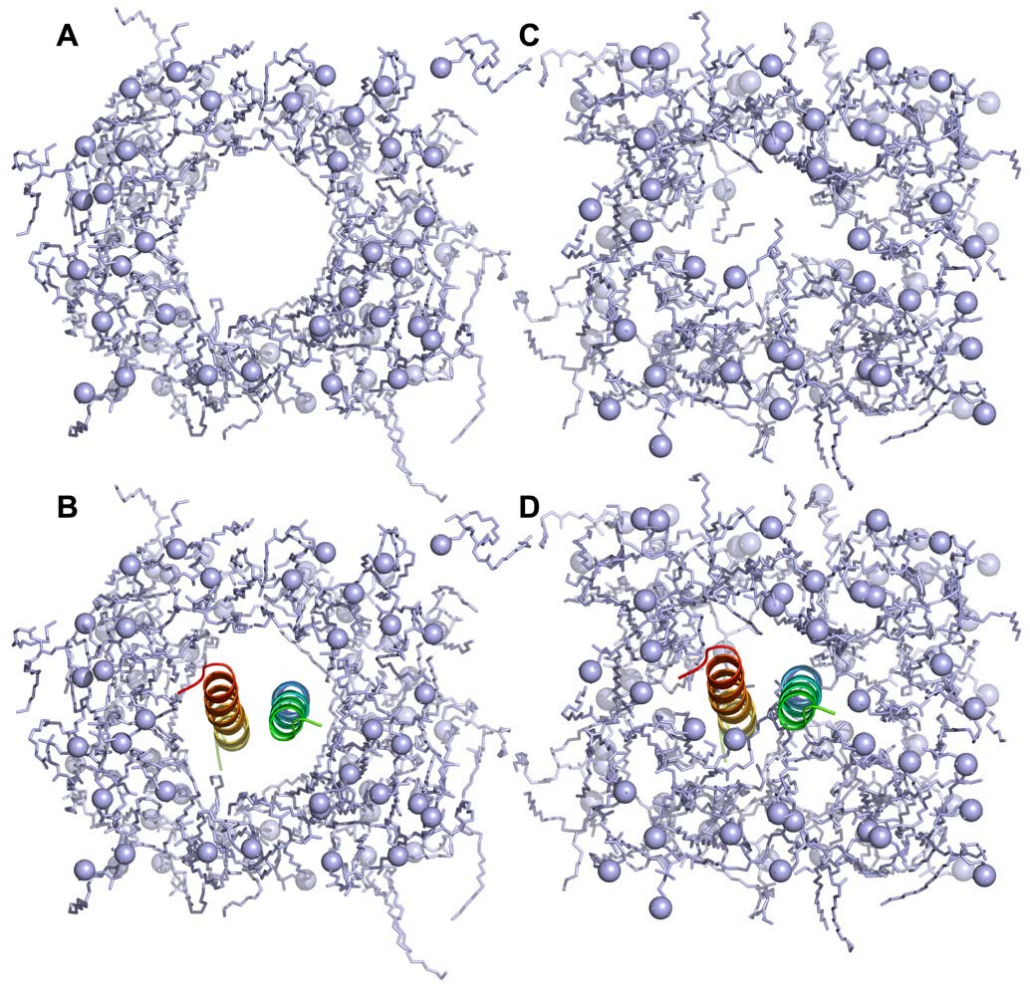
DMPC lipid bilayers generated by (A and B) the insertion method and (C and D) the replacement method for PDB:2HAC.

To generalize and automate the lipid bilayer building method, we have extended and further refined both insertion and replacement methods. Figure 6 shows a clear distinction between insertion and replacement methods. The insertion method creates a hole in a bilayer by applying weak repulsive radial forces during the simulation of the lipid bilayer, and insert a protein into the hole, as shown in Figs. 6A and 6B. By utilizing a library that has various sizes of holes in pre-equilibrated lipid bilayers, the insertion method can generate a lipid bilayer less than a minute. Currently, a library for each lipid molecule (DMPC, DPPC, and POPC) is composed of 90 pre-equilibrated lipid bilayers with a hole of radius from 1 Å to 45 Å for two different number of lipid molecules (128 or 256 lipid molecules) (see Materials and Methods for details). The size of the largest lipid bilayer is 90 Å by 90 Å in the *XY* plane with 256 lipid molecules (128 for each leaflet). Therefore, if a system dimension is larger than 90 Å by 90 Å in *XY*, or the number of lipid molecules in one leaflet is more than 128, Membrane Builder will ask users to select the replacement method. By assuming that the protein has a cylindrical shape, the hole sizes are first estimated from the protein areas in the top and bottom lipid leaflets. Then, the top and bottom lipid leaflets with the estimated hole sizes are selected from the library and the numbers of lipid molecules are reduced to their target numbers determined in Step 3. Therefore, the insertion method is useful for a protein that has a regular and cylindrical shape. Otherwise, the replacement method would be a better choice.

The replacement method distributes lipid-like pseudo atoms around the protein, and substituting each atom with a lipid molecule that is randomly selected from a lipid molecular library. In Membrane Builder, the library is composed of 2,000 different conformations that were gathered from MD simulations of pure lipid bilayers (see Materials and Methods for details). Figure 6D shows a DMPC lipid bilayer generated by the replacement method for PDB:2HAC. Unlike the insertion method, lipid molecules wrap around the protein such that the replacement method can be used for virtually any kinds of membrane proteins, including transmembrane proteins, monotopic proteins, and interfacial peptides. Furthermore, this method should be able to generate a mixed lipid bilayer with slight modification in the lipid substitution process.

There are limitations and advantages in each method. The insertion method can build a lipid bilayer quickly, and the generated lipid bilayer is pre-equilibrated. However, the insertion method has a couple of limitations related to the system size and the protein shape. On the other hand, the replacement method can generate a lipid bilayer for virtually any kinds of shape and size of membrane proteins. However, it takes more time to generate a lipid bilayer, e.g., 5 to 50 minutes depending on the system size, and the generated bilayer is not pre-equilibrated. The library of lipid bilayers with holes and the library of lipid molecules for each lipid type are freely available at the archive on the CHARMM-GUI website.

### Pore Water Generation

Ion channels, transporters, and porins have pores that are large enough to accommodate water molecules. A traditional way of generating the so-called pore water is to build a cylindrical water box, place the box inside the pore, and remove water molecules that overlap with the protein. It is easy to implement and works well for some cases. During the tests of Membrane Builder, however, we have encountered many cases that the traditional method cannot be applied directly. For example, a closed form of mechanosensitive channel (PDB:2OAR) has a pore opened only half of the transmembrane region, and the cross-sectional area profile varies markedly. If the pore were filled by the traditional method, large number of water molecules would be left in the protein exterior near the closed region of the protein. Another example is bacterial porins (e.g., PDB:1PHO) with three pores, so that it is not conceivable to fill the pores automatically with the traditional method.

**Figure 7:**
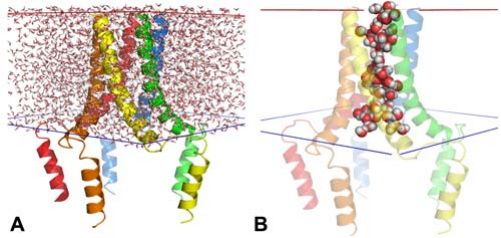
Pore water generation. (A) The transmembrane region of the protein is solvated with a waterbox. (B) Only pore water molecules had remained after pore water generation through high-temperature dynamics.

In order to make the generation process of pore water more general and persuasive, we have developed a new method based on a simple idea that water molecules inside the pore would remain while those outside of the protein would evaporate during a high temperature dynamics. As shown in Table 1, water molecules can access to the pore vestibules in the lipid head group regions, but not into the hydrophobic region due to the restraints for water during equilibration. Therefore, Membrane Builder only solvates the transmembrane region, as shown in Fig. 7A, and runs molecular dynamics at a high temperature (5,000 K) with protein fixed and planar restraint potentials on the top and bottom of the water box to keep pore water molecules in the transmembrane region. Most water molecules that are outside of the protein are quickly removed after a couple of dynamics cycles, as shown in Fig. 7B. However, it is conceivable that a few water molecules may stay near the protein exterior if they interact with protein strongly. To resolve this problem, we have introduced the refinement step of pore water generation in Step 4 to remove those water molecules interactively by selecting residue numbers of water molecules after users' visual inspection. Users can verify this step by visualizing the pore region again after removing selected water molecules.

### Test cases

In order to illustrate and verify the efficiency and applicability of the web-based Membrane Builder, we have generated the protein/membrane complexes for 15 membrane proteins of various sizes and shapes, 12 transmembrane and 3 interfacial proteins (see Table 2). Three kinds of lipid molecules (DMPC, DPPC, and POPC) and two kinds of system shapes (rectangular and hexagonal) were used to build a total of 90 complex systems. The test was facilitated by using pre-oriented protein structures from OPM database [25] and the default values in each step, i.e., 1.5 lipid layers to determine the system size in *XY*, 12 Å water thickness to determine the system size along *Z*, and 0.15 M KCl concentration (the physiological ion concentration), unless specified explicitly. It should be stressed that these default values were elaborated and tested extensively by building the large number of protein/membrane complexes. Table 2 shows the detailed information on the systems with POPC membranes and the full list of 90 complex systems is shown in Table S1 in the Supporting Information. Note that ligands such as ions or small molecules in original PDB structures are not included.

**Table 2:** Testcases and system information with POPC membranes.

PDB ID	System Shape^1^	Numbers of Each Components				Total Atoms	System Size^2^
		Lipid	H_2_O	K^+^	Cl^−^		
1GZM^3^	rect	110	9,173	30	25	47,523	67×74×99
	hexa	79	6,713	24	19	35,977	67×67×99
2OAR^3^	rect	141	16,056	48	48	77,651	84×84×112
	hexa	112	13,582	41	41	66,329	84×84×112
1ZLL^3^	rect	194	12,640	27	47	68,653	89×89×89
	hexa	164	10,544	22	42	58,335	90×90×89
1I78^3^	rect	98	8,174	17	23	44,811	65×65×102
	hexa	86	7,357	25	20	38,222	66×66×102
2GFP^3^	rect	130	8,635	20	26	49,106	80×75×80
	hexa	116	7,946	18	24	45,159	80×80×80
1OKC^3^	rect	125	7,594	14	31	35,605	75×75×82
	hexa	95	5,960	10	27	44,535	73×73×82
1UYN^3^	rect	103	7,995	22	21	42,011	67×67×91
	hexa	87	6,601	20	19	35,681	67×67×91
1H2S^4^	rect	183	14,333	42	36	76,217	108×77×90
	hexa	237	17,772	50	44	93,786	108×108×90
1SU4^4^	rect	452	62,760	178	151	264,614	130×130×147
	hexa	386	53,969	156	129	229,353	130×130×147
1UUN^4^	rect	227	44,526	168	80	214,653	130×130×120
	hexa	187	37,970	151	63	185,973	130×130×120
2A65^4^	rect	303	26,082	69	75	135,400	115×122×95
	hexa	227	21,070	56	62	110,154	115×115×95
1XQ8^4^	rect	243	8,334	18	22	58,978	75×119×64
	hexa	341	11,493	25	29	81,601	119×119×64
2DEO^4^	rect	158	10,367	29	29	55,447	70×80×95
	hexa	156	10,356	29	29	55,146	80×80×95
1D5R^4^	rect	274	18,867	47	58	98,539	100×100×97
	hexa	235	16,288	40	51	85,562	100×100×97
1PHO^4^	rect	230	17,835	63	33	101,695	112×112×82
	hexa	188	15,726	58	28	89,730	113×113×82

1. The system shape in the XY plane: rect for rectangular and hexa for hexagonal.

2. The system size (in Å) is given by *L*_X_×*L*_Y_×*L*_Z_, where *L*_X_, *L*_Y_, and *L*_Z_ correspond the lengths of the system along the *X*, *Y*, and *Z* axes, respectively. In the case of hexagonal systems, *L*_X _( =  *L*_Y_) corresponds to the length of the longest diagonal.

3. The insertion method was used to build protein/membrane complex systems.

4. The replacement method was used to build protein/membrane complex systems.

For given testcases, the whole process of generating a protein/membrane system took 5 minutes to 2 hours depending on the system size. The smallest protein and the largest protein in the testcases are PDB:1UYN (translocation domain of autotransporter NalP) and PDB:1SU4 (calcium ATPase), respectively. We have applied the insertion method for the proteins that have a cylindrical and regular shape, such as PDB:1GZM (bovine rhodopsin), 1ZLL (human phospholamban pentamer), 1I78 (outer membrane protease OmpT), 2GFP (multidrug transporter EmrD), 1OKC (mitochondrial ADP/ATP carrier), 2OAR (mechanosensitive channel of large conductance), 1UYN (translocator domain of autotransporter NalP). The replacement method was used for the other proteins that have rather irregular shape, including interfacial peptides, such as PDB:1H2S (sensory rhodopsin II), 2A65 (a bacterial homolog of Na+/Cl− dependent neurotransmitter transporters), 1SU4 (calcium ATPase), 1PHO (E. Coli porins), 1XQ8 (human micelle-bound alpha-synuclein), 2DEO (membrane protease specific for a stomatin homolog from Pyrococcus horikoshii), PDB:1UUN (main porin from Mycobacteria), and 1D5R (PTEN tumor suppressor). Pore water molecules were generated for PDB:2OAR, 1I78, 1OKC, 1UYN, 1UUN and 1ZLL. There are three membrane proteins such as PDB:1SU4, 1UUN, and 1XQ8 for which the default options could not generate the complex system properly. Both PDB:1SU4 and 1UUN have soluble cellular domains that are much larger than their transmembrane domain. Since Membrane Builder determines the system size in *XY* only based on the size of the transmembrane domain, the system size generated by the default “1.5 lipid layers” was not big enough to accommodate the cellular domain. Therefore, we had to manually adjust their system size. Since the final system size of PDB:1UUN was too big to use the insertion method, we had to use the replacement method, although it has a cylindrical and regular shape. In particular, PDB:1XQ8 has a very elongated conformation along the membrane surface. Since Membrane Builder assumes that the protein shape in the *XY* plane is more or less symmetric, the estimated system size by the default values was much smaller than it should be, such that we had to adjust the system size manually.

Each system was equilibrated for 375 ps in our local machines using the equilibration input files that were generated by Membrane Builder (see Table 1). All the calculations were performed with CHARMM [28], and the all-atom parameter set PARAM22 for proteins [29] including dihedral cross-term corrections (CMAP) [30] and a modified TIP3P water model [31], as well as recently optimized lipid parameters [32]. The NVT dynamics (constant volume and temperature) is used for the first and second steps, and the NPAT (constant pressure, area, and temperature) dynamics for the rest at 303.15K (DMPC and POPC) and 323.15K (DPPC). The equilibrated protein/membrane complexes are freely available at the archive on the CHARMM-GUI website.

## Discussion

We have described the generalized and automated process to build a protein/membrane complex interactively and easily using Membrane Builder in the CHARMM-GUI website. Its efficacy was illustrated by building protein/membrane complexes for 15 membrane proteins of various sizes and shapes. Each and every step is translated into a web interface so that a realistic protein/membrane complex can be generated through a web browser with a graphical user interface. The significance of this work is that the web interface provides not only the intuitive and elegant way to generate the membrane system, but also the detailed input files that are already optimized and tested extensively. We have adopted and further developed two lipid bilayer generation methods, “replacement method” and “insertion method.” The insertion method uses a pre-equilibrated lipid bilayer with a hole of an appropriate size into which a protein is inserted. This method is particularly useful for a protein with cylindrical and symmetrical shape. If a protein has an irregular shape, the replacement method would be a better choice. The replacement method uses a packing of lipid-like pseudo atoms, and replaces them by lipid molecules one at a time. Therefore, it generates a nicely packed lipid bilayer around the protein.

While Membrane Builder currently supports three types of lipid molecules such as DMPC, DPPC, and POPC, we will make more lipid molecules available by generating the necessary lipid libraries. In addition, we will incorporate a method of building mixed lipid bilayers by introducing a step for choosing different types of lipids randomly in the replacement method. Furthermore, Membrane Builder will provide the CPγT (constant pressure, surface tension, and temperature) dynamics [33] to allow the system size along the *X* and *Y* axes to vary during the simulation, and the P2_1_ image transformation [34] to allow the number of lipid molecules in the top and bottom leaflets to vary during the simulation. Although Membrane Builder has been developed to use CHARMM [28] as a MD simulation engine, we will provide the equilibration input files for other MD software such as GROMACS [35] and NAMD [36] in near future so that users can continue the equilibration of the generated membrane system with their own MD software.

## Materials and Methods

### Lipid Bilyaer Library and Lipid Molecule Library

To facilitate the building process of a membrane bilayer using the insertion and replacement methods, the library of lipid bilayers with holes and the library of lipid molecules have been generated. There are 90 pre-equilibrated lipid bilayers with a hole of radius from 1 Å to 45 Å for two different number of lipid molecules (128 or 256 lipid molecules) in the lipid library for DMPC, DPPC, and POPC. Starting from an equilibrated membrane bilayer of each type of lipid, the cylindrical pore has been generated by increasing the pore radius by 1 Å every 50 ps with a cylindrical harmonic restraint at 303.15 K (DMPC and POPC) and at 323.15 K (DPPC). The maximum number of lipid molecules available in each lipid bilayer is 256 (128 for each leaflet), and the largest bilayer size is 90 Å by 90 Å. The lipid molecule library for each type of lipid contains 2,000 different conformations. Lipid molecules are randomly selected from a 2.5 ns trajectory of each lipid bilayer. All the calculations were performed with CHARMM [28], and the all-atom parameter set PARAM22 for proteins [29] including dihedral cross-term corrections (CMAP) [30] and a modified TIP3P water model [31], as well as recently optimized lipid parameters [32].

## Supporting Information

Table S1 (0.23 MB DOC)
