# Supplementary material for: Automated Builder and Database of Protein/Membrane Complexes for Molecular Dynamics Simulations
Source: PLoS One. 2007 Sep 12;2(9):e880. doi: 10.1371/journal.pone.0000880 (PMC1963319; doi:10.1371/journal.pone.0000880)
Supplement: Table S1 — (0.23 MB DOC) [file pone.0000880.s001.doc]

**Automated Builder and Database of Protein/Membrane Complexes for Molecular Dynamics Simulations**

Sunhwan Jo^1^, Taehoon Kim^2^, and Wonpil Im2[[1]](#footnote-2)*

^1^Department of Chemistry,

^2^Department of Molecular Biosciences and Center for Bioinformatics

The University of Kansas

2030 Becker Drive

Lawrence, KS 66047, USA

Keywords: CHARMM-GUI, Insertion method, Replacement Method, Lipid Library, DMPC, DPPC, POPC

Table S1. Testcases and system information.

| PDB ID | System  Shape | Numbers of Each Components | | | | Total Atoms | System Size (Å) |
| --- | --- | --- | --- | --- | --- | --- | --- |
|  |  | Lipid | H_2_O | K^+^ | Cl^-^ |  |  |
| 1GZM^1^ | rectangular | 117 DMPC | 8,712 | 29 | 24 | 45,204 | 65 X 72 X 99 |
|  |  | 113 DPPC | 8,445 | 29 | 24 | 45,287 | 66 X 73 X 99 |
|  |  | 110 POPC | 9,173 | 30 | 25 | 47,523 | 67 X 74 X 99 |
|  | hexagonal | 82 DMPC | 6,433 | 23 | 18 | 34,225 | 65 X 65 X 99 |
|  |  | 81 DPPC | 6,252 | 23 | 18 | 34,536 | 66 X 66 X 99 |
|  |  | 79 POPC | 6,713 | 24 | 19 | 35,977 | 67 X 67 X 99 |
| 2OAR^1^ | rectangular | 150 DMPC | 15,365 | 46 | 46 | 74,380 | 83 X 83 X 112 |
|  |  | 146 DPPC | 15,389 | 47 | 47 | 75,734 | 84 X 84 X 112 |
|  |  | 141 POPC | 16,056 | 48 | 48 | 77,651 | 84 X 84 X 112 |
|  | hexagonal | 119 DMPC | 13,217 | 40 | 40 | 64,266 | 83 X 83 X 112 |
|  |  | 115 DPPC | 13,010 | 40 | 40 | 64,553 | 83 X 83 X 112 |
|  |  | 112 POPC | 13,582 | 41 | 41 | 66,329 | 84 X 84 X 112 |
| 1ZLL^1^ | rectangular | 209 DMPC | 12,326 | 26 | 46 | 66,375 | 88 X 88 X 89 |
|  |  | 202 DPPC | 12,015 | 26 | 46 | 67,040 | 89 X 89 X 89 |
|  |  | 194 POPC | 12,640 | 27 | 47 | 68,653 | 89 X 89 X 89 |
|  | hexagonal | 177 DMPC | 10,472 | 21 | 41 | 57,027 | 88 X 88 X 89 |
|  |  | 171 DPPC | 10,128 | 22 | 42 | 57,341 | 89 X 89 X 89 |
|  |  | 164 POPC | 10,544 | 22 | 42 | 58,335 | 90 X 90 X 89 |
| 1I78^1^ | rectangular | 103 DMPC | 8,291 | 26 | 21 | 41,656 | 63 X 63 X 102 |
|  |  | 101 DPPC | 8,180 | 26 | 21 | 42,299 | 64 X 64 X 102 |
|  |  | 98 POPC | 8,174 | 17 | 23 | 44,811 | 65 X 65 X 102 |
|  | hexagonal | 90 DMPC | 7,045 | 23 | 18 | 36,378 | 64 X 64 X 102 |
|  |  | 88 DPPC | 6,915 | 24 | 19 | 36,810 | 65 X 65 X 102 |
|  |  | 86 POPC | 7,357 | 25 | 20 | 38,222 | 66 X 66 X 102 |
| 2GFP^1^ | rectangular | 137 DMPC | 8,764 | 19 | 25 | 48,237 | 78 X 73 X 80 |
|  |  | 134 DPPC | 8,527 | 19 | 25 | 48,780 | 79 X 74 X 80 |
|  |  | 130 POPC | 8,635 | 20 | 26 | 49,106 | 80 X 75 X 80 |
|  | hexagonal | 123 DMPC | 8,174 | 17 | 23 | 44,811 | 78 X 78 X 80 |
|  |  | 121 DPPC | 7,916 | 18 | 24 | 45,255 | 79 X 79 X 80 |
|  |  | 116 POPC | 7,946 | 18 | 24 | 45,159 | 80 X 80 X 80 |
| 1OKC^1^ | rectangular | 133 DMPC | 7,317 | 13 | 30 | 42,646 | 74 X 74 X 82 |
|  |  | 129 DPPC | 6,992 | 14 | 31 | 34,917 | 75 X 75 X 82 |
|  |  | 125 POPC | 7,594 | 14 | 31 | 35,605 | 75 X 75 X 82 |
|  | hexagonal | 101 DMPC | 5,826 | 10 | 27 | 42,646 | 72 X 72 X 82 |
|  |  | 99 DPPC | 5,684 | 10 | 27 | 42,749 | 73 X 73 X 82 |
|  |  | 95 POPC | 5,960 | 10 | 27 | 44,535 | 73 X 73 X 82 |
| 1UYN^1^ | rectangular | 109 DMPC | 7,691 | 21 | 20 | 40,157 | 65 X 65 X 91 |
|  |  | 105 DPPC | 7,385 | 21 | 20 | 40,027 | 61 X 61 X 91 |
|  |  | 103 POPC | 7,995 | 22 | 21 | 42,011 | 67X 67 X 91 |
|  | hexagonal | 91 DMPC | 6,305 | 19 | 18 | 33,871 | 66 X 66 X 91 |
|  |  | 89 DPPC | 6,197 | 19 | 18 | 34,367 | 66 X 66 X 91 |
|  |  | 87 POPC | 6,601 | 20 | 19 | 35,681 | 67 X 67 X 91 |
| 1H2S^2^ | rectangular | 196 DMPC | 14,522 | 40 | 34 | 75,686 | 107 X 75 X 90 |
|  |  | 190 DPPC | 14,083 | 41 | 35 | 75,643 | 108 X 76 X 90 |
|  |  | 183 POPC | 14,333 | 42 | 36 | 76,217 | 108 X 77 X 90 |
|  | hexagonal | 257 DMPC | 18,340 | 49 | 43 | 94,056 | 107 X 107 X 90 |
|  |  | 248 DPPC | 17,534 | 49 | 43 | 93,552 | 108 X 108 X 90 |
|  |  | 237 POPC | 17,772 | 50 | 44 | 93,786 | 108 X 108 X 90 |
| 1SU4^2^ | rectangular | 508 DMPC | 65,592 | 178 | 151 | 272,486 | 130 X 130 X 147 |
|  |  | 482 DPPC | 62,832 | 178 | 151 | 266,922 | 130 X 130 X 147 |
|  |  | 452 POPC | 62,760 | 178 | 151 | 264,614 | 130 X 130 X 147 |
|  | hexagonal | 434 DMPC | 56,635 | 156 | 129 | 236,839 | 130 X 130 X 147 |
|  |  | 412 DPPC | 54,190 | 156 | 129 | 231,852 | 130 X 130 X 147 |
|  |  | 386 POPC | 53,969 | 156 | 129 | 229,353 | 130 X 130 X 147 |
| 1UUN^2^ | rectangular | 254 DMPC | 47,254 | 168 | 80 | 222,227 | 130 X 130 X 120 |
|  |  | 241 DPPC | 44,657 | 168 | 80 | 216,970 | 130 X 130 X 120 |
|  |  | 227 POPC | 44,526 | 168 | 80 | 214,653 | 130 X 130 X 120 |
|  | hexagonal | 210 DMPC | 40,427 | 151 | 63 | 192,980 | 130 X 130 X 120 |
|  |  | 200 DPPC | 38,221 | 151 | 63 | 188,528 | 130 X 130 X 120 |
|  |  | 187 POPC | 37,970 | 151 | 63 | 185,973 | 130 X 130 X 120 |
| 2A65^2^ | rectangular | 328 DMPC | 26,807 | 67 | 73 | 135,673 | 114 X 121 X 95 |
|  |  | 317 DPPC | 25,831 | 68 | 74 | 135,253 | 115 X 122 X 95 |
|  |  | 303 POPC | 26,082 | 69 | 75 | 135,400 | 115 X 122 X 95 |
|  | hexagonal | 244 DMPC | 25,152 | 54 | 60 | 109,970 | 114 X 114 X 95 |
|  |  | 236 DPPC | 20,834 | 55 | 61 | 109,706 | 115 X 115 X 95 |
|  |  | 227 POPC | 21,070 | 56 | 62 | 110,154 | 115 X 115 X 95 |
| 1XQ8^2^ | rectangular | 263 DMPC | 9,219 | 18 | 22 | 60,105 | 74 X 118 X 64 |
|  |  | 254 DPPC | 8,410 | 18 | 22 | 59,664 | 74 X 119 X 64 |
|  |  | 243 POPC | 8,334 | 18 | 22 | 58,978 | 75 X 119 X 64 |
|  | hexagonal | 372 DMPC | 12,889 | 25 | 29 | 83,991 | 118 X 118 X 64 |
|  |  | 358 DPPC | 11,631 | 25 | 29 | 82,861 | 118 X 118 X 64 |
|  |  | 341 POPC | 11,493 | 25 | 29 | 81,601 | 119 X 119 X 64 |
| 2DEO^2^ | rectangular | 177 DMPC | 11,180 | 29 | 29 | 57,600 | 70 X 80 X 95 |
|  |  | 168 DPPC | 10,428 | 29 | 29 | 56,298 | 70 X 80 X 95 |
|  |  | 158 POPC | 10,367 | 29 | 29 | 55,447 | 70 X 80 X 95 |
|  | hexagonal | 175 DMPC | 11,099 | 28 | 28 | 57,119 | 80 X 80 X 95 |
|  |  | 166 DPPC | 10,291 | 28 | 28 | 55,625 | 80 X 80 X 95 |
|  |  | 156 POPC | 10,356 | 29 | 29 | 55,146 | 80 X 80 X 95 |
| 1D5R^2^ | rectangular | 308 DMPC | 20,101 | 47 | 58 | 101,869 | 100 X 100 X 97 |
|  |  | 293 DPPC | 19,055 | 47 | 58 | 100,477 | 100 X 100 X 97 |
|  |  | 274 POPC | 18,867 | 47 | 58 | 98,539 | 100 X 100 X 97 |
|  | hexagonal | 210 DMPC | 13,714 | 31 | 42 | 71,112 | 90 X 90 X 97 |
|  |  | 251 DPPC | 16,414 | 40 | 51 | 87,000 | 100 X 100 X 97 |
|  |  | 235 POPC | 16,288 | 40 | 51 | 85,562 | 100 X 100 X 97 |
| 1PHO^2^ | rectangular | 247 DMPC | 18,469 | 62 | 32 | 101,921 | 110 X 110 X 82 |
|  |  | 239 DPPC | 17,725 | 62 | 32 | 101,613 | 111 X 111 X 82 |
|  |  | 230 POPC | 17,835 | 63 | 33 | 101,695 | 112 X 112 X 82 |
|  | hexagonal | 201 DMPC | 16,266 | 56 | 26 | 89,872 | 111 X 111 X 82 |
|  |  | 194 DPPC | 15,513 | 57 | 27 | 89,117 | 112 X 112 X 82 |
|  |  | 188 POPC | 15,726 | 58 | 28 | 89,730 | 113 X 113 X 82 |

1. The insertion method was used to build protein/membrane complex systems.

2. The replacement method was used to build protein/membrane complex systems.

1. ^*^ Corresponding author: Phone: (785) 864-1993; Fax: (785) 864-5558; E-mail: wonpil@ku.edu [↑](#footnote-ref-2)
